# A Rare Case of Single-Organ Vasculitis of the Hepatic Artery in a Female Patient: Diagnosis and Management Challenges

**DOI:** 10.7759/cureus.58637

**Published:** 2024-04-20

**Authors:** Julian D Quiceno, Juan F Betancur, Alejandra Solano, Jose D Puerta, Nancy Toro

**Affiliations:** 1 Internal Medicine, Clínica Medellín Grupo QuirónSalud, Medellin, COL; 2 Internal Medicine, Internal Medicine Investigation Unit, Centro de Investigaciones Clínicas SURA, Medellin, COL; 3 Vascular Medicine, Universidad de Antioquia, Medellin, COL; 4 Radiology, Clínica Medellín Grupo QuirónSalud, Medellín, COL

**Keywords:** rheumatoid factor (rf), early onset of rheumatoid arthritis, hepatic artery vasculitis, single organ vasculitis, imaging in vasculitis

## Abstract

This case report describes a rare occurrence of isolated vasculitis of the hepatic artery in a female patient. The patient presented with abdominal pain, fever, and weight loss, and a diagnosis was made through a combination of imaging studies and serological evaluation of systemic vasculitis. The management of this case was challenging because of the involvement of the hepatic artery without any other clinical manifestations of the systemic disease, apart from the presence of rheumatoid factor and anti-citrullinated cyclic peptide. The authors highlight the importance of considering vasculitis as a potential diagnosis in patients with unexplained abdominal pain and fever and the need for a multidisciplinary approach to the management of these patients. This case also emphasizes the potential complications of vasculitis, including aneurysm formation, and the need for close monitoring and follow-up of these patients.

## Introduction

Vasculitis is characterized by the presence of inflammatory leukocytes in the vessel walls with reactive damage to the mural structures. It is a serious and potentially fatal condition that can occur as a primary or secondary process to an underlying disease. The location, size, and type of vasculitis vary depending on the specific type, and prompt recognition and therapy are crucial [1].

Gastrointestinal (GI) tract involvement is often observed in primary vasculitis, particularly in polyarteritis nodosa (PAN), anti-neutrophil cytoplasmic antibody (ANCA)-associated vasculitis, IgA vasculitis, and Takayasu arteritis. GI involvement can occur in approximately one-third of patients with primary vasculitis, whereas it is less common in rheumatoid or lupus vasculitis [2-4]. Isolated vasculitis of the GI tract, without systemic involvement, is rare.

Here, we present an unusual case of isolated vasculitis of the hepatic artery in a female patient.

## Case presentation

A 54-year-old woman with a two-week history of persistent right upper quadrant abdominal pain, accompanied by nausea, fatigue, fever, and unintentional weight loss, was evaluated. The patient was a smoker with a family history of systemic lupus erythematosus in a cousin but had no significant medical history, surgeries, or drug use. Temperature and vital signs were normal on physical examination, which showed unremarkable cardiovascular and respiratory findings. The abdominal examination revealed only minimal tenderness in the epigastrium and right hypochondrium, without any signs of peritoneal irritation (Table 1).

**Table 1:** Laboratory results

Test	Result	Normal Range
Hemoglobin	14.2 g/dL	12-16 g/dL
Leukocyte Count	7.810 /mm^3^	4.500-11.000/mm^3^
Differential Count	Normal	Normal
Platelet Count	235.000	150.000-450.000/mm^3^
CRP (C-reactive protein)	1.05 mg/dL	0.06-0.5 mg/dL
ESR (Erythrocyte Sedimentation Rate)	25 mm/h	0-30 mm/h
Creatinine	0.70 mg/dL	0.51-0.95 mg/dL
AST (Aspartate aminotransferase)	17	5-32 U/L
ALT (Alanine aminotransferase)	11 U/L	0-33 U/L
Alkaline phosphatase	78 U/L	35-104
HBsAg (Hepatitis B surface antigen)	Non-reactive	Non-reactive
Hepatitis C Antibody	Non-reactive	Non-reactive
HIV (Human Immunodeficiency Virus) ELISA	Non-reactive	Non-reactive
VDRL (Venereal disease research laboratory) test	Non-reactive	Non-reactive

**Figure 1:**
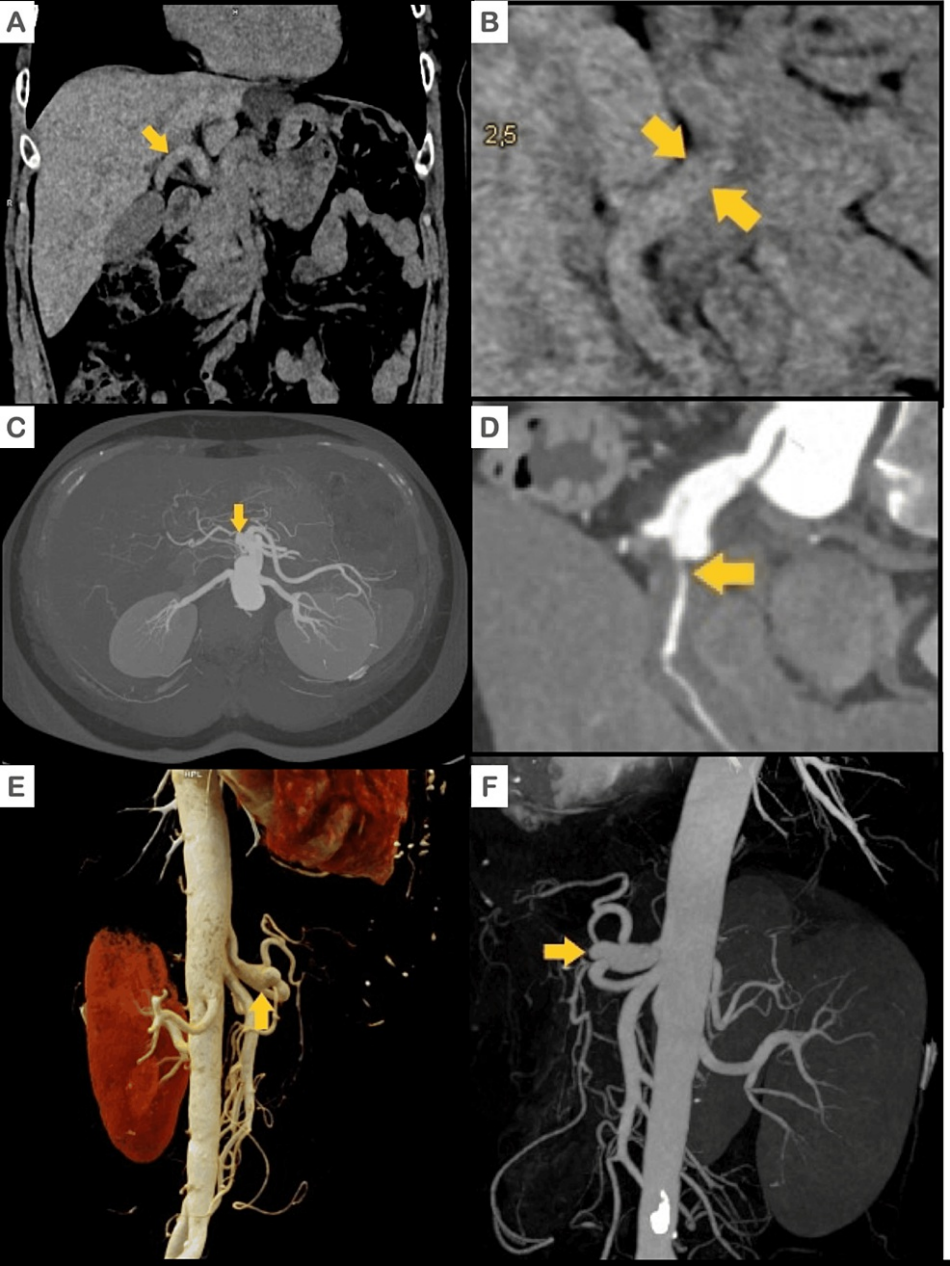
Abdominal CT: Simple, contrasted, and tridimensional reconstruction Simple CT scan of the abdomen. The common hepatic artery with a thickened soft tissue density wall (A: coronal; B: axial). C and D: Computed tomography (CT) of the abdomen revealing dilatation of the distal celiac trunk and minimal lumen of the common hepatic artery (yellow arrow). E and F: Angiotomography with three-dimensional reconstruction depicting a fusiform aneurysm of the celiac trunk and amputation of the common hepatic artery.

Computed tomography (CT) of the abdomen and pelvis (Figures 1A, 1B) revealed circumferential thickening of the wall of the common hepatic artery with a narrowed but filiform lumen and dilatation of the distal celiac trunk before the origin of the common hepatic artery. An angiography tomography scan, including a three-dimensional reconstruction of the neck, chest, and abdomen (as depicted in Figures 1C-1F), was conducted to identify additional vascular irregularities. However, no further abnormalities were detected except for those previously identified using CT.

**Figure 2:**
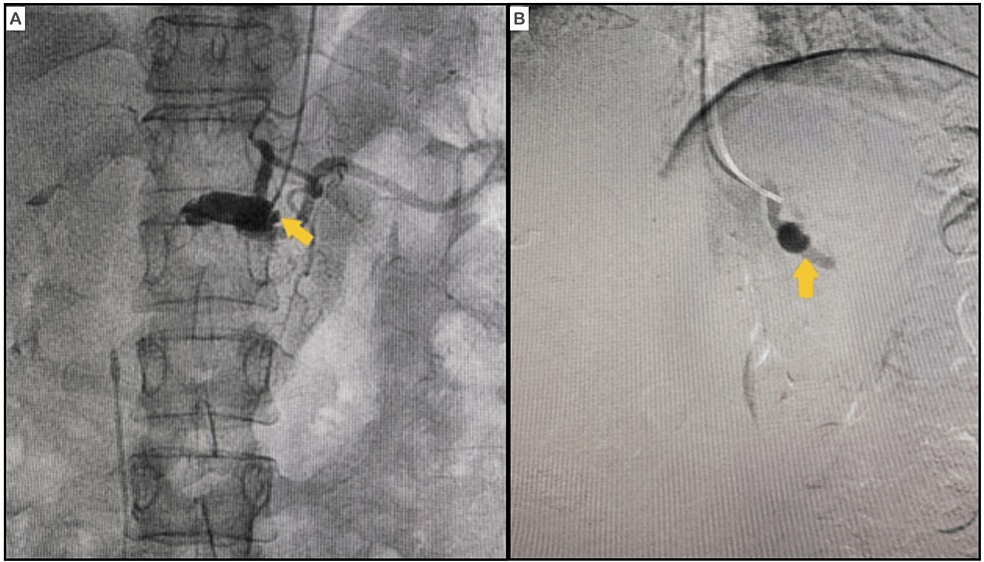
Abdominal arteriogram (A and B) Abdominal arteriogram showing a fusiform aneurysm of the celiac trunk. The hepatic artery exhibited dilatation in the proximal two-thirds and occlusion in the distal portion.

Abdominal arteriography (Figures 2A, 2B) revealed a fusiform aneurysm of the celiac trunk before bifurcation. The proximal two-thirds of the common hepatic artery displayed dilation, while it was completely obstructed in the distal segment, preventing passage of the guidewire or contrast medium. The splenic, left gastric, and superior mesenteric arteries were all normal.

**Figure 3:**
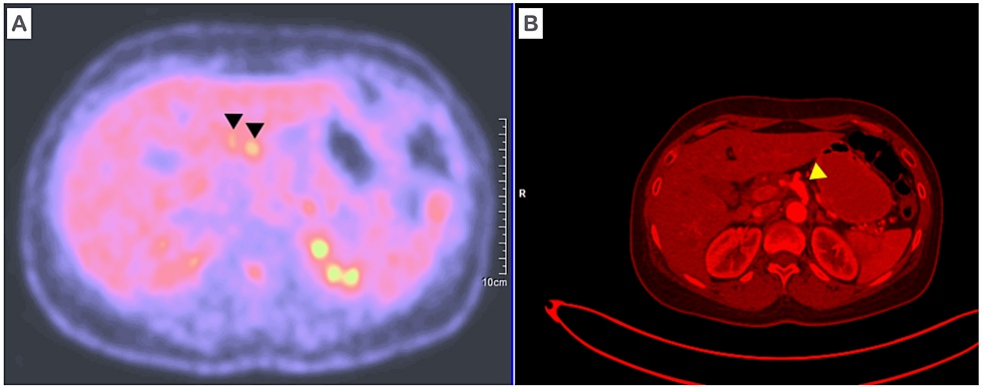
Abdominal 18-fluorodeoxyglucose positron emission tomography (FDG-PET) scan Metabolic hyperactivity in the hepatic artery A. Black head arrows B. Yellow head arrow.

The results of the 18-fluorodeoxyglucose positron emission tomography (FDG-PET) scan revealed hypermetabolism with a maximum standardized uptake value (SUV) of 3.89 in the hepatic artery and hypermetabolism with a maximum SUV of 6.02 in the ascending colon, accompanied by thickening of the colonic wall (Figures 3A, 3B). FDG concentration was found to be within normal limits in all other vascular structures. FDG-PET was performed to detect inflammation in the hepatic artery and ascending colon, differentiating this from other vascular issues through metabolic activity. It guided further diagnostics and treatment by highlighting the disease's extent and potential biopsy sites. Additionally, it refined the differential diagnosis and provided a baseline for monitoring treatment response.

Subsequent to these findings, colonoscopy was performed, which revealed the presence of four small tubular adenomas that were endoscopically removed and confirmed to be low-grade dysplastic on histologic examination. No evidence of vasculitis was noted on endoscopic or histological examinations.

**Figure 4:**
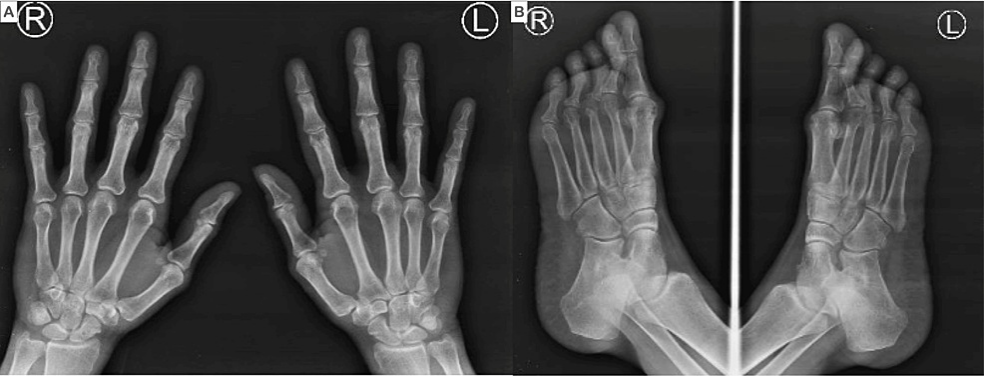
X-rays of hands and feet Normal hands and feet X-rays, without signs suggestive of rheumatoid arthritis.

Serological evaluation for systemic vasculitis was conducted, and it was determined that the patient had negative results for anti-nuclear antibodies (ANA), extractable nuclear antibodies (ENA), anti-DNA-antibodies (anti-DNA), cytoplasmatic and perinuclear anti-cytoplasmatic-antibodies (c-ANCA, p-ANCA), lupus anticoagulant, IgG and IgM anti-cardiolipins, IgG and IgM anti-B2-Glycoproteins, cryoglobulins, and cold agglutinins. The patient's C3 complement level was normal at 119 mg/dL (90-180 mg/dL), and the C4 complement level was normal at 31 mg/dL (10-40 mg/dL). Additionally, the levels of total immunoglobulin IgA, IgM, and IgG were normal, as were the levels of IgG subclasses (IgG1, IgG2, IgG3, and IgG4). Rheumatoid Factor (RF) was positive at 67 units/mL (0 to 14 U/mL), and anti-cyclic citrullinated peptide (anti-CCP) was strongly positive at 285 units/mL (0 to 20 units/mL). However, the patient had no history of morning stiffness, pain, or swollen joints, and radiography of the hands and feet showed no signs of synovitis or bone erosion that could lead to a diagnosis of rheumatoid arthritis (RA) (Figures 4A, 4B).

The patient was diagnosed with single-organ vasculitis (SOV) of the hepatic artery and treated with high-dose methylprednisolone for three days, followed by a steroid-sparing regimen of prednisone and methotrexate 15 mg each week. The patient's symptoms resolved, and she was discharged with continued steroid tapering. One week after discharge, the patient's CRP level and ESR returned to normal levels.

## Discussion

Reaching a diagnosis of vasculitis requires thorough clinical evaluation, a combination of laboratory and advanced imaging studies, and sometimes tissue biopsy, while carefully excluding other differential diagnoses [5]. A multidisciplinary approach involving rheumatologists, gastroenterologists, radiologists, and vascular medicine often facilitates an accurate diagnosis and effective management plan.

The 2012 Chapel Hill Consensus Conference (CHCC) introduced a nomenclature system for the majority of vasculitis forms. The classification of vasculitis primarily depends on the size of the predominant vessels involved, although there may be some overlap in vessel sizes among these diseases [6]. The main categories of vasculitis are as follows (Table 2).

**Table 2:** Chapel Hill consensus conference 2012 classification criteria ANCA: Anti-neutrophil cytoplasmic antibody. Adapted from Chapel Hill Consensus Conference (CHCC) 2012 criteria [5].

Category	Description
Large vessel vasculitis	Takayasu arteritis Giant cell arteritis
Medium vessel vasculitis	Kawasaki disease Polyarteritis nodosa
Small vessel vasculitis	ANCA-associated vasculitis: Granulomatosis with polyangiitis (Wegener granulomatosis) Microscopic polyangiitis Eosinophilic granulomatosis with polyangiitis (Churg-Strauss syndrome). Immune complex small-vessel vasculitis: anti-glomerular basement membrane disease Cryoglobulinemic vasculitis IgA vasculitis (Henoch-Schönlein) Hypocomplementemic urticarial vasculitis (anti-C1q vasculitis)
Variable-vessel vasculitis	Behcet's disease Cogan Syndrome
Single organ vasculitis	Cutaneous leukocytoclastic angiitis Cutaneous arteritis Primary central nervous system vasculitis Isolated aortitis Others
Vasculitis associated with systemic disease	Lupus vasculitis Rheumatoid vasculitis Sarcoid vasculitis Others
Vasculitis associated with probable etiology	Hepatitis C-associated cryoglobulinemic vasculitis Hepatitis B virus-associated vasculitis Syphilis-associated vasculitis Cancer-associated vasculitis Drug-associated vasculitis Others

The differential diagnosis of hepatic artery vasculitis involves a range of conditions that can simulate vasculitis symptoms or imaging findings. These conditions include other vascular, structural, and systemic diseases that affect the abdominal arteries, particularly the celiac trunk and branches. The following is a list of essential differential diagnoses to be considered (Table 3).

**Table 3:** Differential diagnosis of hepatic artery vasculitis

Differential Diagnosis	Characteristics
Atherosclerotic Disease	The most prevalent vascular disease, involves plaque buildup in arterial walls, causing narrowing or blockage. Affects older individuals and those with risk factors such as smoking, dyslipidemia, diabetes, and hypertension.
Median Arcuate Ligament Syndrome (MALS)	Compression of the celiac artery by the median arcuate ligament, causes abdominal pain during exercise in younger, thinner patients. Imaging studies show compression during expiration for diagnosis.
Fibromuscular Dysplasia (FMD)	Affects the medium-sized arteries, causing stenosis, aneurysms, or dissections. Primarily affects women and is diagnosed through angiography, revealing a characteristic "string of beads" appearance.
Aortic Dissection	Severe condition in which the inner layer of the aorta tears, causing blood to separate the layers of the blood vessel wall. If this condition extends to involve the aorta's branches, it can affect the hepatic artery, resulting in acute symptoms.
Aneurysms	Might be due to atherosclerosis, infections, or connective tissue disorders.
Infections	Arteritis is caused by infections in the arterial wall, leading to inflammation. More common in immunocompromised individuals, caused by bacterial, viral, or fungal infections.
Tumors and Cancer	Pancreatic cancer and lymphoma, can directly affect the hepatic artery and indirectly cause compression or invasion of vascular structures.
Radiation Arteritis	Results from prior radiation therapy in the abdominal region, leading to vessel inflammation and damage
Takayasu Arteritis	Arteritis can affect aorta and its branches in younger people and resemble other vasculitis affecting the hepatic artery.

These conditions can present with symptoms similar to those of vasculitis, such as abdominal pain, weight loss, or GI symptoms, and may show overlapping features in imaging studies. Therefore, thorough clinical evaluation, detailed imaging studies, laboratory tests, and histological examination are crucial to differentiate these conditions from vasculitis and guide appropriate treatment.

To address the unique presentation of hepatic artery vasculitis, like in our case, particularly in the context of positive RF and anti-CCP antibodies, it is crucial to tailor the discussion to the specifics observed.

In this case, the category that best fits our patient is SOV, which is characterized by inflammation of the arteries or veins in a single organ, without evidence of systemic involvement [7]. SOV vasculitis is characterized by inflammation of arteries or veins in a single organ without evidence of systemic involvement [7]. The presentation may be unifocal or multifocal, and some patients initially diagnosed with SOV may subsequently develop additional symptoms that reclassify the disease as a systemic vasculitis. Therefore, to accurately diagnose SOV, it is necessary to conduct long-term surveillance for a minimum of six months to confirm the isolated nature of the condition [7].

Our patient's seropositivity for RF and anti-CCP antibodies without the classical clinical or radiographic manifestations of RA presents a peculiar scenario. These markers are closely associated with RA, and anti-CCP is known for its high specificity [8]. Their presence suggests a possible underlying autoimmune process that may not yet manifest as full-blown RA, raising the question of whether SOV could be an early indicator of RA where clinical and radiological changes are pending [8-10].

However, given the isolated vascular involvement and lack of systemic or joint symptoms, it remains speculative to conclude that vasculitis is secondary to early RA. The possibility of false-positive results for these antibodies must also be considered, although the high specificity of anti-CCP for RA makes this less likely [8,10]. The literature describes instances in which anti-CCP positivity precedes the clinical symptoms of RA by years; during this time, critical quantitative and qualitative changes have occurred in anti-CCP proximate to clinical onset on average of 3-5 years prior to the development of clinically apparent RA, with some cases occurring more than 13 years prior [9-12]. This phenomenon, referred to as epitope spreading, is characterized by antibody reactivity to an increasing number of citrullinated autoantigens over time. A limited degree of avidity maturation also occurs before symptom onset, although great variability in anti-CCP avidity exists between patients with RA [10].

This suggests that our patient could be in a pre-clinical phase of RA, with vasculitis as an initial manifestation. However, this hypothesis requires further longitudinal observations for validation.

As extra-articular manifestation, rheumatoid vasculitis typically develops in patients with long-standing seropositive and erosive RA, usually more than 10 years after diagnosis [13]. Few cases have been reported to occur within the first five years of diagnosis, and only one case of vasculitis involving the hepatic artery has been reported in the very early course of the disease, two months after symptom onset [3,14,15]. No cases of rheumatoid vasculitis without signs or symptoms of RA have been previously reported.

In some cases of vasculitis, the clinical-serological approach can be insensitive during both initial evaluation and long-term monitoring, making 18-FDG PET a valuable diagnostic tool [16]. One of the key advantages of PET scans is their ability to detect vasculitis as well as other serious pathologies, such as infections or tumors, particularly in elderly patients with constitutional symptoms who do not exhibit specific clinical features of vasculitis [17]. However, PET scans also have some drawbacks such as high cost, limited availability, and radiation exposure [17].

Management was initiated based on the general principles of SOV of the GI tract and potential early RA involving high-dose steroids and methotrexate.

In a case series conducted by the Mayo Clinic over a period of 12 years, 18 cases of vasculitis affecting the Gl tract were identified [18]. The affected organs included the esophagus, stomach, small or large bowel, peritoneum, appendix, gallbladder, and pancreas, and all patients in this series underwent a negative autoimmune workup. Abdominal pain was the most common symptom, reported in nearly all patients, along with other symptoms, such as abdominal angina, nausea or vomiting, diarrhea, hematochezia, or melena. Of the 18 patients in this case series, ten were treated with prednisone, with a median starting dose of 60 mg/day (30-100 mg/day), while three received methylprednisolone pulse therapy (1 g/day for 3 to 5 days) prior to oral prednisone. The median duration of oral prednisone was 7.5 months (range 1-36), and five patients received additional immunosuppressive therapies such as cyclophosphamide, azathioprine, or methotrexate. Eight of the ten treated patients responded to treatment, with two achieving remission, five experiencing relapse, and three dying (one from hepatic abscesses, one from hepatic infarcts, and one from bowel perforation). Among the eight untreated patients, three achieved remission, one experienced relapse, and four died (three from unknown causes and one from small- and large-bowel ischemia). During the median follow-up period of 10 months, none of the 18 patients showed evidence of systemic vasculitis.

In a case report by Mali et al., a patient with hepatic artery vasculitis was treated with methylprednisolone at a dose of 1000 mg intravenously daily for three days, followed by prednisone, which was subsequently tapered [19]. At the six-month follow-up, the patient reported relief from abdominal pain, and ESR and CRP levels had improved. Abdominal CT revealed a significant decrease in soft tissue density surrounding the common hepatic artery.

Our treatment approach underscores the necessity for tailored treatment protocols that consider the unique aspects of isolated hepatic artery vasculitis and potential side effects of long-term immunosuppression. The absence of a structured follow-up strategy to monitor disease progression, treatment response, and complications highlights a significant gap in the management plan. Given the potential for the evolution of systemic autoimmune diseases and risk of vasculitis-related complications, establishing a comprehensive monitoring framework is essential.

## Conclusions

In our discussion of a case involving isolated vasculitis of the hepatic artery, we navigated through the intricacies of diagnosis, the empirical approach to management, and the critical need for comprehensive follow-up. The rarity of this condition poses significant diagnostic challenges, primarily relying on imaging and serological evaluations, because of the impracticality of obtaining a confirmatory tissue biopsy. The presence of RF and anti-CCP adds complexity, suggesting a potential autoimmune etiology without the clinical manifestation of RA. This serological finding raises questions about vasculitis being an early sign of RA or an unrelated phenomenon, a distinction crucial for guiding long-term management, but remains unresolved. This case underscores the need for a multidisciplinary approach to rare vascular disorders, integrating expertise from rheumatology, gastroenterology, vascular medicine, and radiology to optimize diagnostic accuracy and treatment effectiveness. Future studies should aim to develop clearer diagnostic criteria, evidence-based treatment protocols, and structured follow-up guidelines for isolated vasculitis cases. Enhancing our understanding of such rare conditions through continued research and case documentation will be pivotal for improving patient outcomes and management strategies.
